# Assessment of extraction options for a next‐generation biofuel: Recovery of bio‐isobutanol from aqueous solutions

**DOI:** 10.1002/elsc.202000090

**Published:** 2021-06-18

**Authors:** Chuhan Fu, Zhuoxi Li, Yulei Zhang, Conghua Yi, Shaoqu Xie

**Affiliations:** ^1^ The Gene and Linda Voiland School of Chemical Engineering and Bioengineering Washington State University Pullman WA USA; ^2^ School of Pharmacy Guangzhou Xinhua University Guangzhou P. R. China; ^3^ School of Chemistry & Chemical Engineering South China University of Technology Guangzhou P. R. China

**Keywords:** biofuels, energy requirement, extraction, isobutanol, separation

## Abstract

Isobutanol is a widely used platform compound and a raw material for synthesizing many high value‐added compounds. It also has excellent fuel properties and is an ideal gasoline additive or substitute with a very broad development space. Isobutanol production by biological fermentation has the advantages of a comprehensive source of raw materials, low cost, environmental protection, and sustainability. However, it also has disadvantages such as many impurities, low isobutanol concentration, and difficulty separating the water + isobutanol azeotrope. Thus, it is necessary to explore an appropriate downstream separation process for the water + isobutanol azeotrope. K_2_CO_3_ with a strong salting‐out effect was used as the salting‐out agent, and the salting‐out of isobutanol from aqueous solutions was investigated at 298.15 K. The effect of the initial salt concentration in the aqueous solution, the recovery of isobutanol, and the effect of dehydration were investigated in detail. The e‐NRTL‐RK model was employed to generate the binary parameters for isobutanol and water, and electrolyte pair parameters for water/isobutanol and ions to reproduce the phase diagram with high accuracy. The processes of solvent extractive distillation, and salting‐out + distillation were simulated by Aspen Plus. The energy consumptions for the solvent‐based and salting‐out‐based processes were compared. The salting‐out + distillation process turned out to be more energy‐saving than the solvent extraction process.

**Abbreviations**
a: short for anion
a, b, c, d, e: parameters
Aϕ: the Debye‐Huckel constant for osmotic coefficients
*b*
: the molality of salt in the aqueous phase
c: short for cation
C_I_
: the initial salt concentration
D: the dielectric constant
d_0_
: the mixed solvent density
e: the electronic charge
G, τ and g: energy parameters
g^ex^
: the excess Gibbs energy of electrolyte systems
g^ex*,lc^
: a short‐range interaction contribution
g^ex*,pdh^
: a long‐range interaction contribution
g^ex,lc^
: the contribution from the local composition (lc) interactions
g^ex,pdh^
: the contribution arising from long‐range ion‐ion interactions using Pitzer‐Debye‐Huckel (pdh) equations
i: refer to ions
i and j: refer to different species
*I_x_*
: the ionic strength parameter
k: Boltzmann constant
Keq: The equilibria constant
m: short for solvent (molecule, water or isobutanol)
subscript m: isobutanol
M: the molar mass of the salt in g/mol
*M_s_*
: the molecular weight of the solvents
*m_salt_*
: the mass of the anhydrous salt
*m_water_*
: the mass of water in the whole system
N_A_
: Avogadro's number equal to 6.0232 x 10^23^ mole^‐1^
R: the universal gas constant (8.314 J mol^−1^ K^−1^)
S_21_
: solubility of isobutanol
T: the temperature (K)
subscript w: water
*x*
: the mole fraction
Z_i_
: the absolute value of the charge on the species of i
α: the non‐randomness factor that can be set as a fixed value
γ_i_
: the activity coefficient of component i in the mixture
ε_w_
: the dielectric constant of water; and r_i_ is the Born radius of segment species i
ρ: the closest distance parameter
τ: the dimensionless interaction parameter
ω_31_
: the mass fraction of salt in the aqueous phase
ω_11_
: the mass fraction of water in the aqueous phase
ω_21_
: the mass fraction of isobutanol in the aqueous phase

## 1 INTRODUCTION

The use of fossil fuels emits a lot of greenhouse gas carbon dioxide, and produces some pollution smoke; thus, threatening global sustainable development. In order to slow the pace of climate change, countries in Europe and the United States are vigorously developing biofuels [1]. Biofuels refer to the solid, liquid, or gas fuel made from biomass or extracted, which can replace gasoline and diesel made from petroleum and is an important direction for the development and utilization of renewable energy [2]. The so‐called biomass refers to various organisms produced through photosynthesis using the atmosphere, water, land, etc., that is, all living organic substances that can grow. It includes plants, animals, and microbes. Unlike traditional fuels such as oil, coal, and nuclear power, these emerging fuels are renewable [3].

Biofuels, in a narrow sense, only refers to liquid biofuels, mainly including bioethanol, biodiesel, biojet, etc. [4, 5, 6]. Bioisobutanol has attracted more and more attention due to its wide application and excellent fuel performance [7]. Isobutanol is a raw material for the synthesis of many high value‐added compounds [8]. It is also a compound with excellent fuel performance. Compared with ethanol, isobutanol has higher energy density, lower oxygen content, and lower hygroscopicity, making it an ideal gasoline additive or substitute [9, 10]. However, there are many problems in the production of bioisobutanol, such as low fermentation concentration [11], many impurities [12], the formation of azeotropes with water [13], resulting in high separation costs. Thus, the development of an efficient and low‐cost downstream separation process has become one of the keys to improving the competitiveness of bio‐based isobutanol [14].

Isobutanol fermentation is a complex biotechnological process during which *Escherichia coli* [15]*, Escherichia coli* [16]*, Saccharomyces cerevisiae* [17]*, Corynebacterium glutamicum* [18], and other bacteria/microorganisms convert sugars to isobutanol, ethanol, carbon dioxide, and other metabolic byproducts. However, when isobutanol is produced by fermentation, lower pH, and products will inhibit fermentation [19, 20, 21]. In the traditional fermentation process, the titer of isobutanol is generally very low, ranging from 0.1 g to 21.2 g/L [21, 22, 23]. Isobutanol fermentation is a relatively complicated process [24, 25]. Firstly, intermediates such as pyruvate, and 2‐ketoacids, are produced in the acid production period. Then acid‐based intermediates were converted to isobutyraldehyde. At last, isobutanol is produced in the solvent production period. Product inhibition makes it difficult to achieve the full utilization of high‐concentration sugars in the fermentation process so that the solvent yield is low, and there are many impurities in the fermentation broth. The low solvent concentration will increase the separation cost [26]. Therefore, it is particularly important to remove the solvents that are toxic to cells from the fermentor.

The separation of low‐concentration isobutanol by traditional distillation requires a lot of energy and cannot achieve real‐time control and productivity of fermentation. To achieve in‐situ separation of isobutanol during the production process, remove product inhibition, and increase product concentration, separation techniques such as adsorption [27], vacuum evaporation [7, 14], liquid‐liquid extraction [28], pervaporation [29, 30], and gas stripping [31] emerged. Although the adsorption method is simple to operate, it has the problems of adsorbent saturation and desorption, and it is not easy to realize the feeding operation. The selectivity of absorbents to isobutanol is poor, and the absorbents are easy to be polluted by the impurities in the fermentation broth. Vacuum evaporation can recover isobutanol at low temperatures. However, extra energy consumption is needed for the flashing process. The liquid‐liquid extraction can easily separate the product based on the principle of two‐phase insolubility. However, the extractant is often toxic to cells, while the non‐toxic liquid extractant usually has low selectivity to isobutanol. The pervaporation has good selectivity and simple operation, but the fouling issue is an avoidable problem. Compared with liquid‐liquid extraction methods, it is susceptible to the fermentation broth. Contamination and clogging of particles are challenging for applying pervaporation in industry. Compared with other separation methods, gas stripping coupled fermentation is harmless to the culture medium. However, there is no obvious gas, such as carbon dioxide and hydrogen generated during the fermentation process. An external gas source is a driving force for the in‐situ removal of isobutanol.

**PRACTICAL APPLICATION**

After the isobutanol fermentation broth is pretreated in the mashing tower, isobutanol + water azeotrope is obtained at the top of the tower. The isobutanol + water azeotrope requires further separation for the purification of isobutanol and consumes a lot of energy. In this work, K_2_CO_3_ with a strong salting‐out effect was used as the salting‐out agent to break the isobutanol + water azeotrope and reduce the energy requirements for the separation of isobutanol + water azeotrope and isobutanol purification. The processes of solvent extractive distillation, and salting‐out + distillation were simulated by Aspen Plus. The energy consumptions for the solvent‐based and salting‐out‐based processes were compared. The salting‐out + distillation process turned out to be more energy‐saving than the solvent extraction process.

**FIGURE 1:**
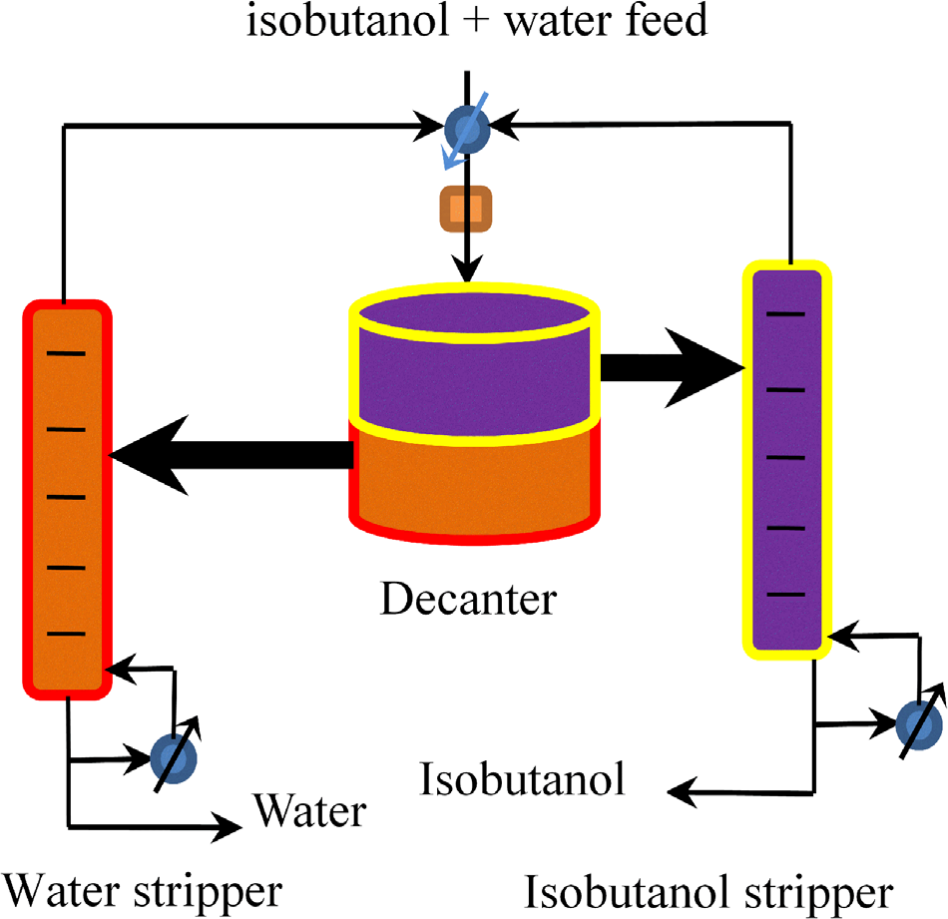
Process flow diagram for the separation of isobutanol + water azeotrope. Adapted with permission from Elsevier [32]

After the isobutanol fermentation broth is pretreated in the mashing tower, 65.6 wt% isobutanol is obtained at the top of the tower, which requires further purification and consumes a lot of energy. The subsequent separation of isobutanol and water mixture can be achieved through two strippers injunction with a decanter because the removal of water from isobutanol and the recovery of isobutanol from the water must be removed in the form of isobutanol + water azeotrope, as shown in Figure 1 [32, 33]. The isobutanol and water mixture is sent to a decanter for phase separation, generating an organic solvent‐rich phase and an aqueous phase. A small amount of isobutanol is dissolved in the aqueous phase. By contrast, A small amount of water is also dissolved in the organic phase. The organic phase from the decanter is sent to the isobutanol stripper to remove water at the top of this stripper. Thus, isobutanol is purified at the bottom of the isobutanol stripper. On the other hand, the aqueous phase from the decanter is sent to the water stripper to remove isobutanol as well. Thus, isobutanol from the aqueous phase is recovered at the top of the water stripper. All the isobutanol + water azeotrope from two strippers is cooled down and send back to the decanter again for the phase separation.

A third component (called extractant or solvent) is usually added to the raw material solution to change the relative volatility of the original components and obtain the separation of an azeotrope. But the boiling point of the extractant is much higher than the boiling point of each component in the raw material liquid, and the extractant does not form an azeotrope with the original components. The salting‐out effect can also effectively eliminate the existence of azeotrope and reduce the energy consumption for purification due to the selective solvation, namely hydration of the inorganic ions. [26, 34–39] In the previous study, the effects of extractant types, concentration and extraction temperature on the extraction efficiency of isobutanol from aqueous solutions were studied when cyclopentanol, tert‐pentanol, n‐valeraldehyde and isooctyl alcohol were used as extractants.[28] The salting‐out extraction of isobutanol from aqueous solutions was also investigated by employing nine salts (K_4_P_2_O_7_·3H_2_O, K_2_HPO_4_·3H_2_O, K_3_PO_4_·3H_2_O, K_2_CO_3_, K_2_SO_4_, KCl, Na_2_CO_3_, Na_2_SO_4_, NaCl) as salting‐out agents and three organic solvents (2‐ethyl‐1‐hexanol, cyclopentanol, 2‐methyl‐2‐butanol) as extractants at 298.15 K [40]. Higher recovery of isobutanol and dehydration ratio was achieved simultaneously with the K_2_CO_3_ as the salting‐out agent. In this work, K_2_CO_3_ with a strong salting‐out effect was used as the salting‐out agent, and the salting‐out of isobutanol from aqueous solutions was investigated at 298.15 K. The effect of the initial salt concentration in the aqueous solution, the recovery of isobutanol, and the effect of dehydration were investigated in detail. The e‐NRTL model was used to correlate the LLE of our systems. [41] Two separation processes, including solvent extractive distillation, and salting‐out + distillation were simulated and analyzed by Aspen Plus for the assessment of extraction options for the recovery of bio‐isobutanol from aqueous solutions.

## 2 MATERIALS AND METHODS

### 2.1 Materials

The reagents used in our study were all analytical grade without further purification. Their purities and manufacturer are shown in Table S1. The purity of isobutanol was checked by gas chromatography. The purities of salts were checked by flame atomic absorption spectrometry (FAAS) using KCl as the standard. They were all in accord with the marked mass concentration. All the chemicals were used as received. The electrical conductivity of deionized water at 293.15 K was lower than 1.5·10^–4^ S·m^–1^.

### 2.2 Experimental method

In this experiment, a 65.6 wt% isobutanol aqueous system was prepared as a simulated water + isobutanol azeotrope to study the salting‐out effect of different salting‐out agents on isobutanol. Then, a salt was added to the 65.6 wt% aqueous system until the system phase separated.

Salt was added gradually until the salt is saturated and precipitate to study the influence of different salt concentrations on the salting‐out effect. The salting‐out procedure was carried out in a 20 mL headspace bottle, sealed with a polytetrafluoroethylene/silica gel pad and then vigorously shaken for 1 h, and placed in an environment of 298.15 K for 24 h to achieve equilibrium [35].

### 2.3 Analytical method

Gas chromatography was used to determine the phase composition of the equilibrium system. The gas chromatograph (Techcomp GC7900, China) is equipped with a 2 m(L) × 3 mm(ID) × 5 mm(OD) Porapak Q 80–100 mesh packed column with a carrier gas (H_2_) flow rate of 30 mL/min and a thermal conductivity detector (TCD). All the samples were measured twice and then averaged to ensure the accuracy of the experimental results.

The salt content in the organic phase is determined by FAAS at the wavelength of 766.5 nm, and the detection method is an external standard method. A nitric acid (1:1, V/V with water) aqueous solution and a 10 g/L cesium nitrate solution were added to the samples for pretreatment [42, 43]. All samples were measured twice to get the average value. The salt concentrations of the aqueous phase were computed by difference.

## 3 MODELING OF THE LIQUID‐LIQUID EQUILIBRIA

It is very important to select an appropriate activity coefficient model for the calculation of liquid‐liquid equilibrium in the extraction system. For example, Arce et al. [44] regressed the phase equilibrium data of quaternary system composed of 1‐octanol+2‐methoxy‐2‐methylbutane + water + methanol at 25°C by using Wilson, universal quasi chemical (UNIQUAC) and non‐random two‐liquid (NRTL) activity coefficient models to obtain the binary interaction parameters of these components. However, for phase equilibrium data of different systems composed of the electrolytes, the electrolyte Non‐Random Two‐Liquid (eNRTL) model presented as a comprehensive excess Gibbs energy expression is more commonly used to represent the liquid‐phase nonideality for aqueous and mixed‐solvent electrolyte systems over the entire concentration range from pure solvents to saturated solutions or fused salts. [41] At present, there are few studies on the activity coefficient models of highly‐solubility salt systems. The utility of the model is demonstrated with liquid−liquid equilibrium of several mixed solvent electrolyte systems composed of water + 2‐propanol +highly soluble salts. [42] In light of the large ionic species in the ternary system, the e‐NRTL model [41] was used again to correlate the LLE of the water + isobutanol +highly soluble salts. Aspen Plus V9 with the Unsymmetric e‐NRTL property method (eNRTL‐RK) was employed for the data regression.

The e‐NRTL model for the excess Gibbs free energy contains two contributions: a long‐range interaction contribution (g^ex*,pdh^) and a short‐range interaction contribution(g^ex*,lc^), as shown in Equation (1).

(1) $$\frac{g^{ex*}}{RT}=\frac{g^{ex*,\;pdh}}{RT}\;+\frac{g^{ex*,\;lc}}{RT}$$

where g^ex^ is the excess Gibbs energy of electrolyte systems, g^ex,lc^ is the contribution from the local composition (lc) interactions, g^ex,pdh^ is the contribution arising from long‐range ion‐ion interactions using Pitzer‐Debye‐Huckel (pdh) equations, R is the universal gas constant (8.314 J mol^−1^ K^−1^), *T* is the temperature (K), and the notation “*” denotes the unsymmetric convention. [41] Accordingly, the Equation (1) leads to the following expression for activity coefficients,

(2) $$\ln\left(\gamma_{i}\right)=\ln\left(\gamma_{i}^{pdh}\right)\;+\ln\left(\gamma_{i}^{lc}\right)$$

where γ_i_ is the activity coefficient of component i in the mixture.

The pdh equations for the long‐range contribution are used to express the excess Gibbs free energy,

(3) $$\begin{array}{ccc} \frac{g^{ex*,\;pdh}}{RT} & = & -\left(\sum_{k}x_{k}\right)\\ & & \times \;{\left(\frac{1000}{M_{s}}\right)}^{1/2}\left(\frac{4A_{\emptyset }I_{x}}{\rho }\right)\ln\left(1+\rho I_{x}^{1/2}\right) \end{array}$$

where *I_x_* is the ionic strength parameter, *M_s_* is the molecular weight of the solvent *s*, and Aϕ is the Debye‐Huckel constant for osmotic coefficients, as shown in the following,

(4) $$I_{x}=\left(\frac{1}{2}\sum Z_{i}^{2}x_{i}\right)\;$$

(5) $$A_{\emptyset }=\frac{1}{3}\;{\left(\frac{e}{\sqrt{DkT}}\right)}^{3}\sqrt{\frac{2\pi d_{0}N_{A}}{1000}}$$

For the Equations ((3), (4), (5)), i refer to ions, *Z*_i_ is the absolute value of the charge on the species of i, *x* is the mole fraction, and ρ is the closest distance parameter. Moreover, N_A_ is Avogadro's number equal to 6.0232 × 10^23^ mole^−1^, k is Boltzmann constant, e is the electronic charge, *d*_0_ is the mixed solvent density, and *D* is the dielectric constant, respectively. The densities of the solvents are from Aspen Plus. Accordingly, Equation (3) leads to the following expression for activity coefficients,

(6) $$\begin{array}{ccc} & & \ln\;\left(\gamma_{i}^{pdh*}\right)=\;-{\left(\frac{1000}{M_{s}}\right)}^{1/2}\\ & & A_{\emptyset }\left[\left(\frac{2Z_{i}^{2}}{\rho }\right)\ln\left(1+\rho I_{x}^{1/2}\right)+\frac{\left(Z_{i}^{2}I_{x}^{1/2}-2I_{x}^{3/2}\right)}{\left(1+\rho I_{x}^{1/2}\right)}\right] \end{array}$$

The short‐range interaction contribution is based upon the NRTL model (NRTL local interaction contribution),

(7) $$x_{cm}+x_{am}+\;x_{mm}=\;1$$

(8) $$x_{ma}+\;x_{ca}=\;1$$

(9) $$x_{mc}+\;x_{ac}=\;1$$

where *c* is short for cation, *a* is short for anion, and m is short for solvent (molecule, water, or isobutanol).

*G*, *τ*, and *g* are energy parameters [41] and given by,

(10) $$\tau_{ji}=\frac{\left(g_{ji}-g_{ii}\right)}{RT}\;$$

(11) $$\begin{array}{c} \tau_{ij}=a_{ij}\;+\frac{b_{ij}}{T-273.15\;K}+e_{ij}\left(\frac{T_{ref}-T}{T}+\ln\frac{T}{T_{ref}}\right) \end{array}$$

(12) $$\alpha_{ij}=c_{ij}+d_{ij}\left(T-273.15\;K\right)$$

(13) $$G_{ji}=\;exp\left(-\alpha \tau_{ji}\right)$$

where *τ* is the dimensionless interaction parameter, *i* and *j* refer to different species, *a*, *b*, *c*, *d*, *e* are parameters, and α is the non‐randomness factor that can be set as a fixed value. However, *e* and *d* were set to 0 in this study. The calculated excess Gibbs energy values from Equation (13) can be used to calculate both the overall excess Gibbs energy for the short‐range interaction contribution (Equation (14)) and the activity coefficients for each component.

(14) $$\begin{array}{ccc} \frac{g^{ex*,lc}}{RT} & = & x_{m\;}\;\left(x_{cm\;}+x_{am\;}\right)\tau_{ca,m}+x_{c\;}x_{mc\;}Z_{c}\tau_{m,ca}\\ & & +x_{a\;}x_{ma\;}Z_{a\;}\tau_{m,ca}+x_{c\;}\left(Z_{c}\tau_{m,ca}+G_{cm}\tau_{ca,m}\right)\\ & & -x_{a\;}\left(Z_{a}\tau_{m,ca}+G_{ma}\tau_{ca,m}\right) \end{array}$$

(15) $$\begin{array}{ccc} \ln\;\gamma_{c}^{lc*} & = & \frac{x_{m}^{2}\tau_{cm}G_{cm}}{{\left(x_{c}G_{cm}+x_{a}G_{am}+x_{m}\right)}^{2}}\\ & & -\;\frac{Z_{a}x_{a}\tau_{ma}x_{m}G_{ma}}{{\left(x_{c}+x_{m}G_{ma}\right)}^{2}}+\frac{Z_{c}x_{m}\tau_{mc}G_{mc}}{\left(x_{a}+x_{m}G_{mc}\right)}\\ & & -\;Z_{c}\tau_{mc}-G_{cm}\tau_{cm} \end{array}$$

(16) $$\begin{array}{ccc} \ln\;\gamma_{a}^{lc*} & = & \frac{x_{m}^{2}\tau_{am}G_{am}}{{\left(x_{c}G_{cm}+x_{a}G_{am}+x_{m}\right)}^{2}}\\ & & -\frac{Z_{c}x_{c}\tau_{mc}x_{m}G_{mc}}{{\left(x_{a}+x_{m}G_{mc}\right)}^{2}}+\frac{Z_{a}x_{m}\tau_{ma}G_{ma}}{\left(x_{c}+x_{m}G_{ma}\right)}\\ & & -Z_{a}\tau_{ma}-G_{am}\tau_{am} \end{array}$$

(17) $$\begin{array}{ccc} \ln\;\gamma_{m}^{lc} & = & x_{cm}\;\tau_{cm}+x_{am}\tau_{am}+\frac{Z_{c}x_{c}G_{mc}\tau_{mc}x_{a}}{{\left(x_{a}+G_{mc}x_{m}\right)}^{2}}\\ & & +\;\frac{Z_{a}x_{a}G_{ma}\tau_{ma}x_{c}}{{\left(x_{c}+G_{ma}x_{m}\right)}^{2}}-\frac{x_{c}x_{m}G_{cm}\tau_{cm}}{{\left(x_{c}G_{cm}+x_{a}G_{am}+x_{m}\right)}^{2}}\\ & & -\;\frac{x_{a}x_{m}G_{am}\tau_{am}}{{\left(x_{c}G_{cm}+x_{a}G_{am}+x_{m}\right)}^{2}} \end{array}$$

where *x*_ij_ is given by,

(18) $$x_{im}=\frac{x_{i}G_{im}}{\left(x_{a}G_{am}+x_{c}G_{cm}+x_{m}\right)}\;$$

(19) $$x_{ac}=\frac{x_{a}}{\left(x_{a}+x_{m}G_{mc,ac}\right)}\;$$

(20) $$x_{ca}=\frac{x_{c}}{\left(x_{c}+x_{m}G_{ma,ca}\right)}\;$$

Fortunately, there are following relationships between different *τ* parameters,

(21) $$\tau_{am}=\tau_{cm}\;=\tau_{ac,m}\;$$

(22) $$\tau_{mc.ac}=\tau_{ma,ca}\;=\tau_{m,ca}\;$$

The mixed solvent (water and isobutanol) was used. Thus, the Born correction that uses the dielectric constants for the long‐range interactions was adapted to calculate the unsymmetric pdh formula,

(23) $$\frac{\Delta G^{born}}{RT}=\frac{Ne^{2}}{2kT}{\left(\frac{1}{\varepsilon_{\delta }}-\frac{1}{\varepsilon_{\omega }}\right)}_{i}\frac{x_{i}Z_{i}^{2}}{r_{i}}10^{-2}$$

where *ε*_w_ is the dielectric constant of water; and *r*_i_ is the Born radius of segment species *i*. The detailed expression for the activity coefficient of segment species i can be found on the reference [41].

For the correlation of the tie‐line data, the equilibria chemistry included in this analysis is provided in Table S2. We only regressed water–ion pair and isobutanol–ion pair because the equilibria constant is very large. The undissociated salt could be negligible so that water–undissociated salt and isobutanol–undissociated salt parameters were kept at zero. The equilibria constant is calculated by,

(24) $$\ln\left(\mathrm{Keq}\right)=A$$

where the concentration basis for Keq is mole fraction. Furthermore, the dielectric constants of isobutanol and water at 25°C were set as 18 and 80.4.

There are six interaction parameters (*τ*_mca_, *τ*_cam_, *τ*_wca_, *τ*_caw_, *τ*_mw_, and *τ*_wm_) and three non‐randomness factors (α_wca_, α_mw_, and α_mca_) for the eNRTL‐RK model. But in this study, the α_mw_ was set to 0.3 and α_wca_ was set to 0.2 [41, 45], and m and w represent isobutanol and water, respectively. Other parameters were obtained from the regression of the LLE data. The default objective function in our study was the maximum likelihood objective function.

## 4 RESULTS AND DISCUSSION

The experimental tie‐line data of (water + isobutanol+ K_2_CO_3_) ternary system at 298.15 K and *p* = 0.1 MPa were shown in Table **1**. The initial salt concentration is defined as shown in the following formula,

(25) $$C_{I}=\frac{m_{salt}\left(g\right)\times 1000}{m_{salt}\left(g\right)+m_{water}\left(g\right)}$$

where *m_salt_* is the mass of the anhydrous salt, and *m_water_* is the mass of water in the whole system. When the initial concentration of K_2_CO_3_ added to the isobutanol + water mixture was greater than 50 g/kg, a new organic phase and a new aqueous phase formed. The upper liquid phase in a headspace bottle was mainly composed of water and isobutanol. The lower liquid phase was mainly composed of water, isobutanol, and K_2_CO_3_. When the initial concentration of K_2_CO_3_ is relatively low, the separation efficiency of isobutanol is not obvious due to the high water content of the organic phase and the high isobutanol content of the aqueous phase. When the initial concentration of K_2_CO_3_ increased from 50 to 500 g/kg, the water content of the organic phase decreased from 13.84 to 2.85 wt%, and the isobutanol content of the aqueous phase decreased from 3.90 wt% to less than 0.00 wt%, suggesting that with the increase of the initial concentration of K_2_CO_3_, the separation efficiency of isobutanol also increased.

**TABLE 1:** Experimental tie‐line data of (water + isobutanol+ K_2_CO_3_) ternary system at 298.15 K and *p* = 0.1 MPa^a^

	Organic phase			Aqueous phase		
C_I_	Water	Isobutanol	K_2_CO_3_	Water	Isobutanol	K_2_CO_3_
(g/kg)	ω_12_ × 100	ω_22_ × 100	ω_32_ × 100	ω_11_ × 100	ω_21_ × 100	ω_31_ × 100
50	13.84	86.16	0.00	89.38	3.90	6.72
100	12.22	87.78	0.00	85.23	1.92	12.85
150	10.81	89.19	0.00	80.58	0.95	18.47
200	9.51	90.49	0.00	75.97	0.41	23.62
250	8.26	91.74	0.00	71.17	0.19	28.64
300	7.01	92.99	0.00	66.62	0.08	33.30
350	5.88	94.12	0.00	62.06	0.00	37.94
400	4.77	95.23	0.00	57.57	0.00	42.43
450	3.78	96.22	0.00	53.06	0.00	46.94
500	2.85	97.15	0.00	48.56	0.00	51.44

ω_ij_ = mass fraction of one component in the aqueous or organic phase (subscript i = 1,2,3 represent water, isobutanol, and salt, respectively; subscript j = 1,2 represent the aqueous phase and organic phase, respectively).

^a^ Standard uncertainties u are u(ω_water_) = 0.003, u(ω_isobutanol_) = 0.003, u(ω_32_) = 0.00003, u(ω_31_) = 0.003, u(T) = 0.05 K, u(p) = 0.0015 MPa.

It can also be seen from Table **1** that with the decrease of the mass fraction of water in the organic phase, the mass fraction of K_2_CO_3_ in the organic phase can be negligible. When the initial concentration of K_2_CO_3_ is greater than 350 g/kg, the content of isobutanol in the aqueous phase is about 0.00 wt%, which can also be negligible for the salting‐out process.

**FIGURE 2:**
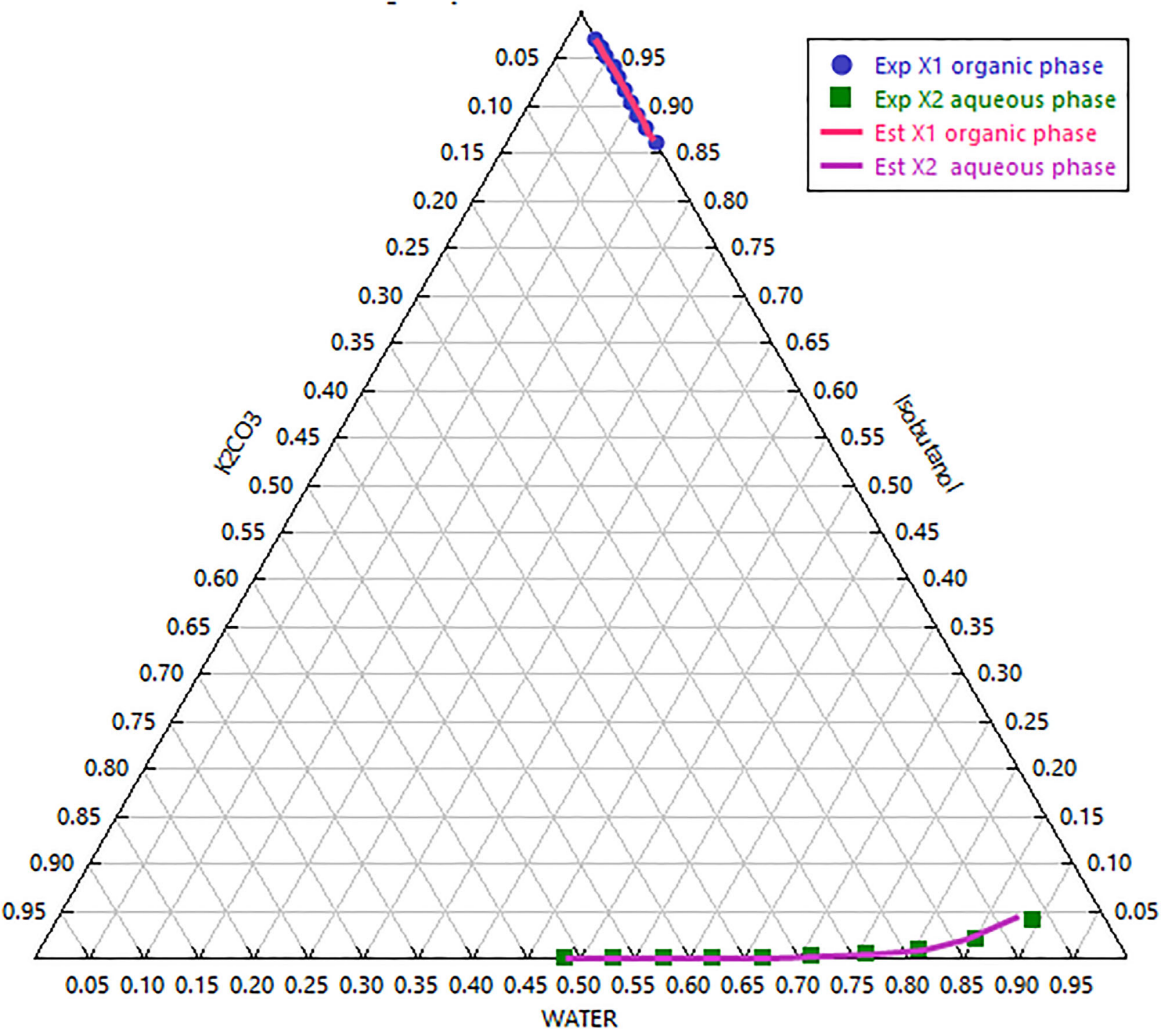
Experimental and estimated triangular phase diagram for isobutanol + K_2_CO_3_ + water systems at T = 298.15 K. Experimental tie‐lines (symbol) and the calculated ones(lines)

The interaction parameters of the eNRTL‐RK model can be obtained according to the experimental data. The binary parameters for isobutanol and water, or electrolyte pair parameters for water/isobutanol and ions, are reported in Table S3. Different non‐randomness factors for alcohols and electrolytes in relevant experimental LLE data are different. The tie‐lines data were correlated successfully by the generalized e‐NRTL‐RK model using the parameters reported in Table S3. Moreover, to show the reliability of the e‐NRTL‐RK model in correlating the tie‐lines data, the experimental phase diagram and estimated phase diagram are shown in Figure 2. Thus, the LLE data for the water + isobutanol+ K_2_CO_3_ ternary system at 298.15 K and *p* = 0.1 MPa can be reproduced with excellent accuracy by using the e‐NRTL‐RK model.

**FIGURE 3:**
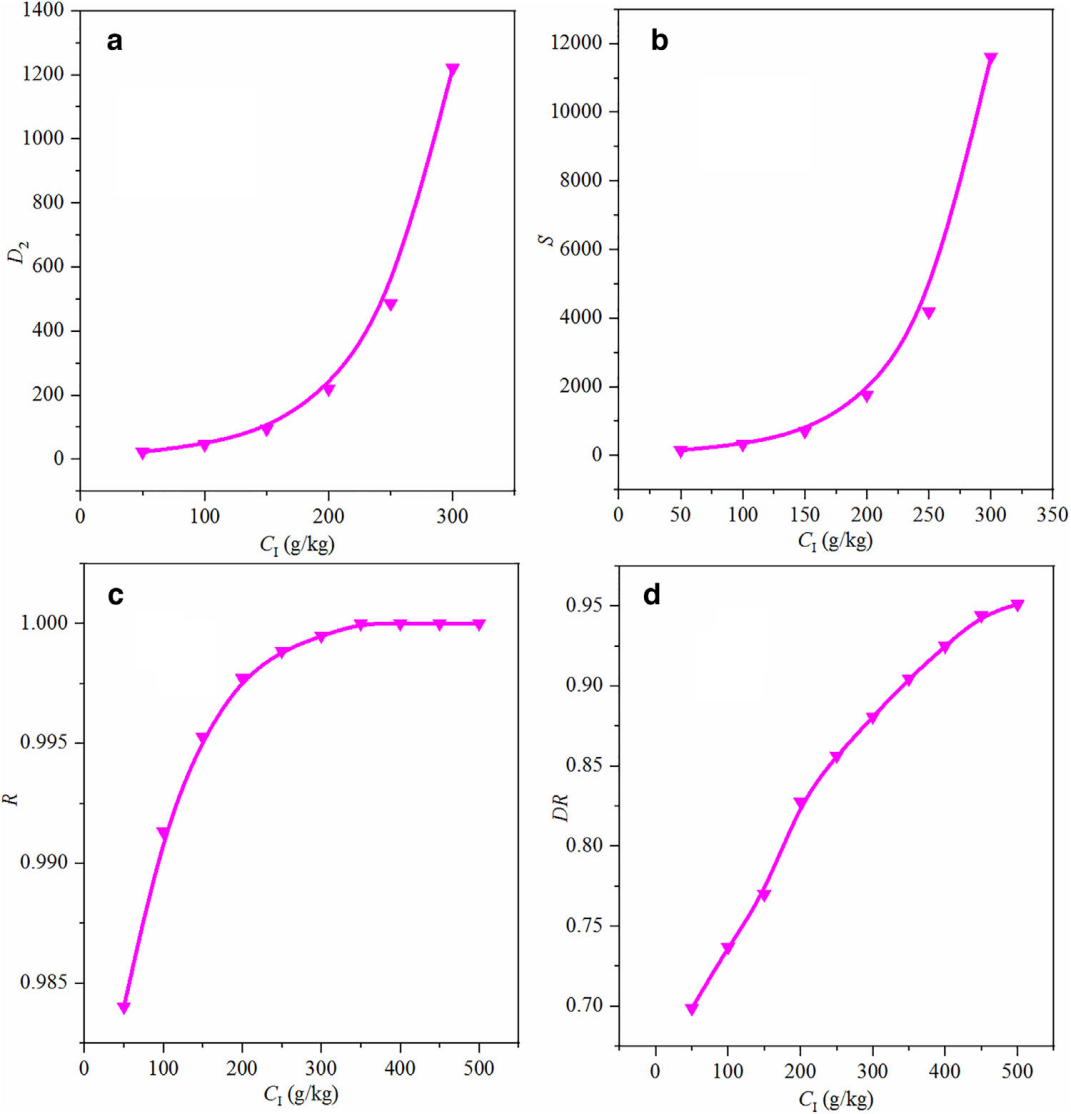
Effects of salt concentration on the distribution coefficient (a), selectivity coefficient (b), recovery (c), and dehydration ratio (d) of isobutanol for the water + isobutanol + inorganic salt system at 298.15 K

It can be seen from Figure 3a that the concentration of salt has a significant effect on the partition coefficient of isobutanol. As the initial molar salt concentration increases, more isobutanol was repelled into the organic phase, and more water molecules were retained in the aqueous phase. Therefore, the partition coefficient of isobutanol gradually increases with increasing salt concentration.

Figure 3b shows the effects of salt's concentration on the selectivity coefficient of isobutanol when the temperature is 298.15 K. K_2_CO_3_ shows a strong selectivity to isobutanol, which is more conducive to the separation and purification of isobutanol. As the initial molar salt concentration increases, the partition coefficient of isobutanol gradually increases, and the partition coefficient of water gradually decreases, so the selectivity coefficient of isobutanol also increases.

Figure 3c shows the effect of salt concentration on the recovery of isobutanol at 298.15 K. As the initial molar salt concentration increased, the recovery rate of isobutanol increased significantly. K_2_CO_3_ has a strong salting‐out effect and shows a high recovery for isobutanol. When the initial salt concentration of K_2_CO_3_ is 350 g/kg, all isobutanol in the aqueous phase can be extracted into the organic phase, and the recovery of isobutanol is as high as 100%. When K_2_CO_3_ is used as the salting‐out agent, and the recovery rate of isobutanol is 100%, the amount of the salting‐out agent used in the salting‐out method is a little higher than that of the salting‐out extraction method (250 g/kg) [40].

Figure 3d shows the effect of the concentration of salt on the dehydration ratio of isobutanol when the temperature is 298.15 K. The results showed that with the increase of the initial molar salt concentration, the dehydration ability of the salting‐out agent to isobutanol was significantly enhanced, and the higher salt concentration was beneficial to the removal of water in the organic phase. When K_2_CO_3_ was used as a salting‐out agent, the dehydration ratio of the organic phase can reach up to 94.4%. The higher dehydration ratio makes the subsequent separation and purification steps lower energy consumption.

In our previous study, the relationship between the molality of salt in the aqueous phase and the solubility of alcohols in the aqueous phase was correlated successfully [38, 42–46]. This solubility equation can effectively associate the salt with the phase equilibrium composition, giving the salting‐out effects of different salting‐out agents [47, 48, 49, 50, 51, 52].

In the experiment of separating isobutanol from aqueous solution by salting‐out, the solubility equation was introduced again to make a more comprehensive evaluation of the salting‐out effects. The solubility of isobutanol in the aqueous phase is defined as shown in the formula,

(26) $$s_{21}=\frac{\omega_{21}}{\omega_{11}}\;\times 100$$

Where ω_21_ is the mass fraction of isobutanol in the aqueous phase, and ω_11_ is the mass fraction of water in the same phase

The molality of salt in the aqueous phase is defined as follows:

(27) $$b\;=\frac{\omega_{31}\times 1000}{M\times \omega_{11}}\;$$

Where *ω*_11_ is the mass fraction of water in the aqueous phase, ω_31_ is the mass fraction of salt in the aqueous phase, and *M* is the molar mass of the salt in g/mol.

**FIGURE 4:**
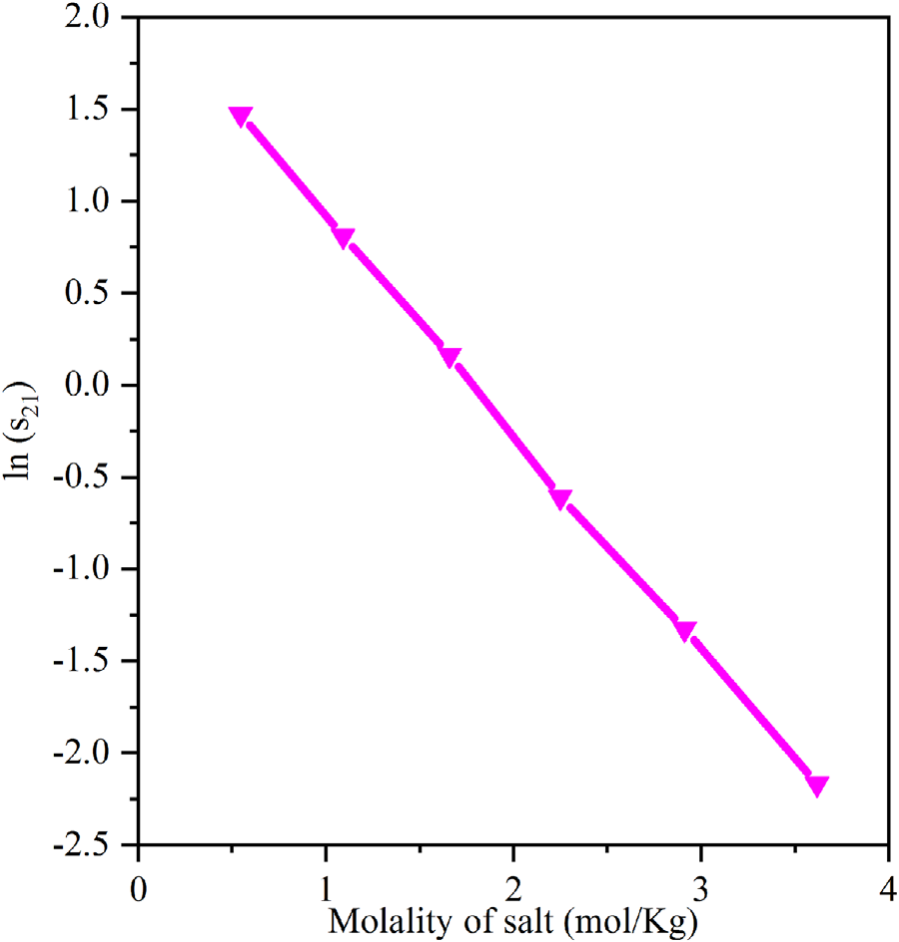
Relation between ln(s_21_) and the molality of salt

Figure 4 plots the curves of lns_21_ and the molality of salt. It can be seen that there is a good linear relationship between the solubility of isobutanol in the aqueous phase and the molality salt in the aqueous phase. The linear relationship is shown in formula (28).

(28) $$\ln\;S_{21}=\alpha b+\beta$$

The parameters of the solubility equation are listed in Table S4.

Cyclopentanol, 2‐methyl‐2‐butanol, and n‐valeraldehyde were used as extraction agents to study the effects of extraction agent type, concentration and extraction temperature on the extraction efficiency of isobutanol from dilute aqueous solutions. [28] Moreover, nine salts (K_4_P_2_O_7_·3H_2_O, K_2_HPO_4_·3H_2_O, K_3_PO_4_·3H_2_O, K_2_CO_3_, K_2_SO_4_, KCl, Na_2_CO_3_, Na_2_SO_4_, NaCl) and three organic solvents (2‐ethyl‐1‐hexanol, cyclopentanol, 2‐methyl‐2‐butanol) were also used to recover isobutanol from dilute aqueous solutions at 298.15 K. In this study, a new process based on the determination of the salt + isobutanol + water system was proposed to separate isobutanol from the isobutanol + water system. Thus, the solvent extractive distillation separation process of isobutanol, the salting‐out extractive distillation separation process of isobutanol, and the salting‐out + distillation separation process of isobutanol from the isobutanol+ water system were simulated by Aspen Plus. Specified streams and parameters for the two processes are shown in Table S5. Analysis of energy consumption was compared for the assessment of extraction options for the recovery of bio‐isobutanol from aqueous solutions.

**FIGURE 5:**
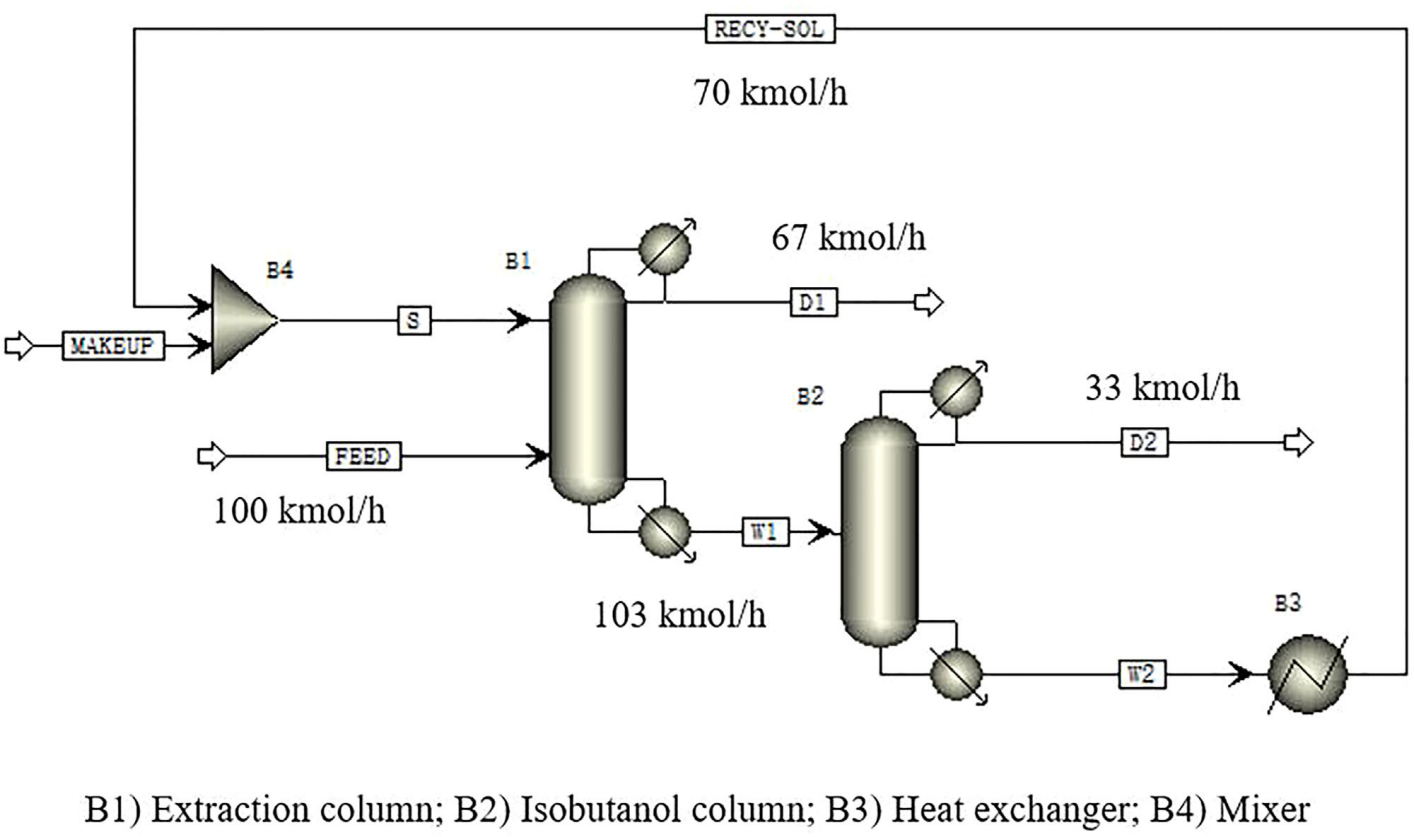
Extractive distillation flowsheet for isobutanol‐water system

An extractive distillation flowsheet for the separation of isobutanol from isobutanol‐water system is shown in Figure 5. B1 is an extractive distillation column. The mixture of isobutanol and water is sent to the middle part of the extractive column, and the extractant is sent to the upper part of the extractive column. The feeding conditions were set as follows: the feed stream was a mixture of water and isobutanol, the molar ratio of water to isobutanol was 67:33 [53], and the molar flow rate was 100 kmol/h at 90℃. The stream S is the extractant, namely isooctanol, and the molar flow rate is 70 kmol/h. Water is condensed and taken at the top of the extraction column, and a mixture of isooctanol, isobutanol and a small amount of water is obtained at the bottom of the column after the extractive distillation. The mixture enters the isobutanol column B2. Isobutanol is condensed and taken at the top condenser of the isobutanol column, and isooctanol is taken at the bottom of this column after the solvent recycling process. The extractant is sent to the mixer for the second run after cooled down in the heat exchanger. The operating parameters for the extractive distillation process are shown in Table S6.

In the extractive distillation process, the efficient and energy‐saving operation should meet the following conditions: (1) the energy consumption of the reboiler of each distillation column should be as low as possible; (2) the composition of the target product in the distillate from the top of the recovery column should be as high as possible. After the extractive distillation, the mass fraction of isobutanol is 99.99%, and 32.8 kmol/h isobutanol was obtained. According to energy consumption analysis, the sum of energy requirements for the reboiler of the extraction column and isobutanol column is 5.59 MJ/kg isobutanol, with 4.26 MJ/kg isobutanol for the extraction column and 1.33 MJ/kg isobutanol for the isobutanol column.

When the salting‐out extraction is used, the organic phase still contains ternary components of organic solvent, isobutanol and water, and the separation steps are complicated. There is energy consumption for the recycling of the solvent. Thus, the salting‐out extraction process is not discussed in this paper.

The salt content of the organic phase in our previous experiment was quantified by FAAS. The results show that the salt content in the organic phase is 0, and all the salts were retained in the aqueous phase. After being fully extracted by the salting‐out effect, the organic phase contains only a small amount of water and isobutanol, and the aqueous phase contains only salt ions and water. Afterward, further dehydration of the organic phases is required to obtain high‐purity isobutanol.

**FIGURE 6:**
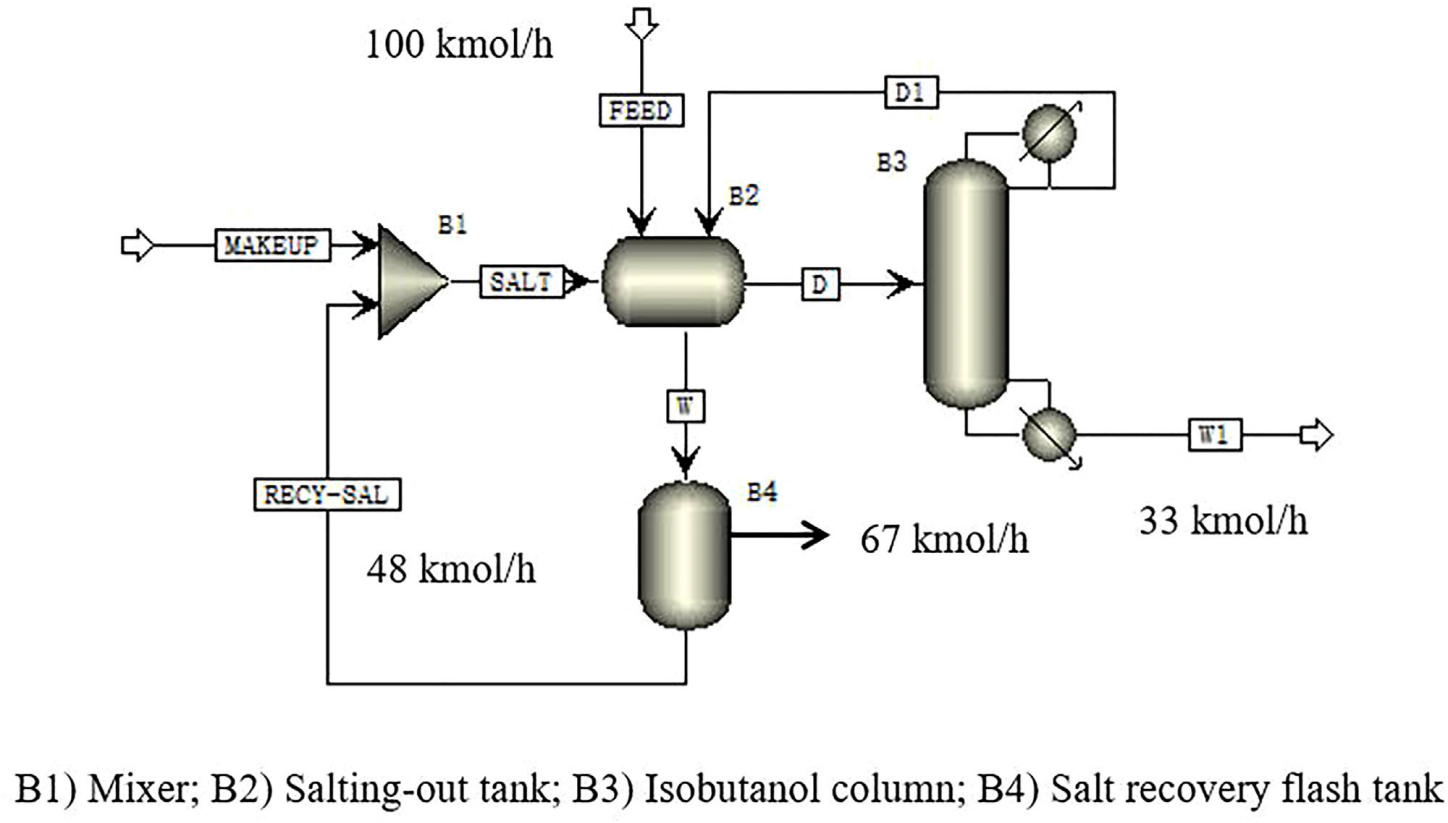
The salting‐out + distillation flowsheet for the isobutanol‐water system

The salting‐out + distillation process to separate isobutanol from the water + isobutanol mixture is shown in Figure 6. The 60 wt% K_2_CO_3_ aqueous solution and isobutanol‐water azeotrope are mixed in the salting‐out tank B2 for phase splitting. The feeding conditions are as follows: the feed stream was a mixture of water and isobutanol, the molar ratio of water to isobutanol was 67:33, and the molar flow rate was 100 kmol/h at 298.15 K. The salting‐out agent is a 60 wt% K_2_CO_3_ aqueous solution with a molar flow rate of 48 kmol/h. The upper organic phase is a mixture of isobutanol and water, which is sent to the isobutanol column B3 for further separation. The distillate in the isobutanol column is composed of water and isobutanol, which is recycled into the salting‐out tank for phase separation. The residual water in the organic phase is removed in this manner. Isobutanol is obtained at the bottom of the isobutanol column. The aqueous phase is a saline solution, which is sent to the salt recovery flash tank B4 to evaporate the excess water. Then the concentrated K_2_CO_3_ solution is sent to the salting‐out tank for the next run.

With the salting‐out + distillation process, the mass fraction of isobutanol achieved ∼100%. The energy requirement for isobutanol recovery, in this case, is only 2.34 MJ/kg isobutanol. The salting‐out + distillation process turned out to be more energy‐saving than the solvent extraction process. The separation of the water + isobutanol azeotrope is simplified by the salting‐out of K_2_CO_3_. No extractant recovery column is needed so that equipment and operating costs are reduced.

## 5 CONCLUDING REMARKS

In this paper, salting‐out was used to separate isobutanol from an isobutanol + water mixture. The experimental results show that in the salting‐out system, as the initial molar salt concentration increases, the partition coefficient, selectivity coefficient, recovery, and dehydration ratio of isobutanol all increase accordingly. After being separated by salting‐out, the organic phase only contains water and isobutanol, and all the salts are retained in the aqueous phase, which also makes the subsequent separation easier. The e‐NRTL‐RK model was employed to generate the binary parameters for isobutanol and water, and electrolyte pair parameters for water/isobutanol and ions to reproduce the phase diagram with high accuracy. The processes of solvent extractive distillation, and salting‐out + distillation were simulated by Aspen Plus. The energy consumptions for the solvent‐based and salting‐out‐based processes were compared. The salting‐out + distillation process turned out to be more energy‐saving than the solvent extraction process.

## CONFLICT OF INTEREST

The authors have declared no conflict of interest.

## Supporting information

Supporting information.

## Data Availability

The data that supports the findings of this study are available in the supplementary material of this article.
