# Supplementary material for: Assessment of extraction options for a next‐generation biofuel: Recovery of bio‐isobutanol from aqueous solutions
Source: Eng Life Sci. 2021 Jun 18;21(10):653–65. doi: 10.1002/elsc.202000090 (PMC8518583; doi:10.1002/elsc.202000090)
Supplement: Supplementary file 1 — Supporting information. [file ELSC-21-653-s001.docx]

**Assessment of extraction options for a next-generation biofuel: recovery of bio-isobutanol from aqueous solutions**

Chuhan Fu,^a^ Zhuoxi Li,^b^ Yulei Zhang,^c^ Conghua Yi,^c^* Shaoqu Xie ^a^*

*^a^*The Gene and Linda Voiland School of Chemical Engineering and Bioengineering, Washington State University, Pullman, WA 99164, USA

*^b^* School of Pharmacy, Xinhua College of Sun Yat-sen University, Guangzhou, China

^c^ School of Chemistry & Chemical Engineering, South China University of Technology, No. 381 Wushan Road, Guangzhou 510640, PR China

**AUTHOR INFORMATION**

Corresponding Authors

Conghua Yi: chyi@scut.edu.cn

Shaoqu Xie: [xieshaoqu@foxmail.com](mailto:xieshaoqu@foxmail.com), [xieshaoqu@163.com](mailto:xieshaoqu@163.com), [shaoqu.xie@wsu.edu](mailto:shaoqu.xie@wsu.edu)

**Table S1.** Main reagents.

| Reagents | purity | manufacturer |
| --- | --- | --- |
| Isobutanol | 99.0% | Damao chemical reagent factory, Tianjin, China |
| K_2_CO_3_ | 99.0% | Guangdong Guanghua Sci-Tech Co., Ltd., (Guangzhou, China) |
| KCl | 99.8% | Shanghai Aladdin Bio-Chem Technology Co.,LTD. |

**Table S2.** Chemistry for the data regression.

| Specification type | Stoichiometry | Equilibrium constant (A) |
| --- | --- | --- |
| Equilibrium | K_2_CO_3_⇌2K^+^+CO_3_^2-^ | 45 |

**Table S3.** Values of parameters for the six interaction parameters of e-NRTL-RK model for isobutanol (m) + salt (ca) + water (w) systems at T = 298.15 K ^a^.

| Species | Binary parameters or electrolyte pair parameters | | | | | |
| --- | --- | --- | --- | --- | --- | --- |
|  | $a_{ij}$ | $b_{ij}$ | $c_{ij}$ | $a_{ji}$ | $b_{ji}$ | $c_{ji}$ |
| Water(i)+ isobutanol (j) | 4.224 | 80.049 | 0.3 | -0.442 | 173.802 | 0.3 |
| Water(i)+K_2_CO_3_(j) | 13.337 | -533.336 | 0.2 | 4.734 | -2997.656 | 0.2 |
| Isobutanol (i)+ K_2_CO_3_ (j) | 12.082 | 809.336 | 0.296 | 1.056 | -4129.082 | 1.050E-05 |

^a^ Standard uncertainties u are u(T) = 0.05 K.

**Table S4.** The solubility correlation constants for the investigated salting-out systems.

| Salt | α | β | R^2^ |
| --- | --- | --- | --- |
| K_2_CO_3_ | -1.185 | 2.1075 | 0.9995 |

**Table S5.** Specified streams and parameters for two processes.

| Steams and parameters | Solvent extractive distillation | Salting-out + distillation |
| --- | --- | --- |
| Feed (kmol/h) | 100.00 | 100.00 |
| Solvent (kmol/h) | 70.00 | - |
| K_2_CO_3_ (kmol/h） | - | 48 |
| Isobutanol in feed (mole fraction) | 0.33 | 0.33 |
| Water in feed (mole fraction) | 0.67 | 0.67 |

**Table S6.** Equipment and operating parameters for the extractive distillation process.

| Extraction column |  | Isobutanol column |  |
| --- | --- | --- | --- |
| number of theoretical stages | 35 | number of theoretical stages | 15.00 |
| Stage 1 pressure (atm) | 1.00 | Stage 1 pressure (atm) | 1.00 |
| Feed stream (above-stage) | 31 | W1 (above-stage) | 10 |
| Solvent (above-stage) | 2 |  |  |
| Reflux ratio | 3.50 | Reflux ratio | 0.60 |
